# Natural Pigments Recovery from Food By-Products: Health Benefits towards the Food Industry

**DOI:** 10.3390/foods13142276

**Published:** 2024-07-19

**Authors:** Daniela Magalhães, Ricardo Gonçalves, Cristina V. Rodrigues, Helena R. Rocha, Manuela Pintado, Marta C. Coelho

**Affiliations:** CBQF—Centro de Biotecnologia e Química Fina, Laboratório Associado, Escola Superior de Biotecnologia, Universidade Católica Portuguesa, Rua Diogo Botelho 1327, 4169-005 Porto, Portugal; dmagalhaes@ucp.pt (D.M.); rdgoncalves@ucp.pt (R.G.); civrodrigues@ucp.pt (C.V.R.); mhrocha@ucp.pt (H.R.R.); mpintado@ucp.pt (M.P.)

**Keywords:** natural pigments, food by-products, extraction technologies, bioactivities, food applications, health benefits

## Abstract

Given the health risks associated with synthetic colorants, natural pigments have emerged as a promising alternative. These renewable choices not only provide health benefits but also offer valuable technical and sensory properties to food systems. The effective application of natural colorants, however, requires the optimization of processing conditions, exploration of new sources, and development of novel formulations to ensure stability and maintain their inherent qualities. Several natural pigment sources have been explored to achieve the broad color range desired by consumers. The purpose of this review is to explore the current advances in the obtention and utilization of natural pigments derived from by-products, which possess health-enhancing properties and are extracted through environmentally friendly methods. Moreover, this review provides new insights into the extraction processes, applications, and bioactivities of different types of pigments.

## 1. Introduction

Over the last decades, the food processing industry has expanded to fulfil the demand for food associated with the growing global human population [1]. The consumption of by-products may be a solution to satisfy the population’s demands. The large amount of food by-products produced during food processing has been explored for the recovery of valuable components, for both economic and environmental reasons [2]. While the food industry has traditionally focused on the development of synthetic colorants for their stability, attractive colors, and low cost, natural food pigments are gradually gaining popularity. This shift is attributed to changing consumer lifestyles and increased concerns regarding potential adverse health effects and environmental damage caused by synthetic colorants/pigments [3].

**Figure 1:**
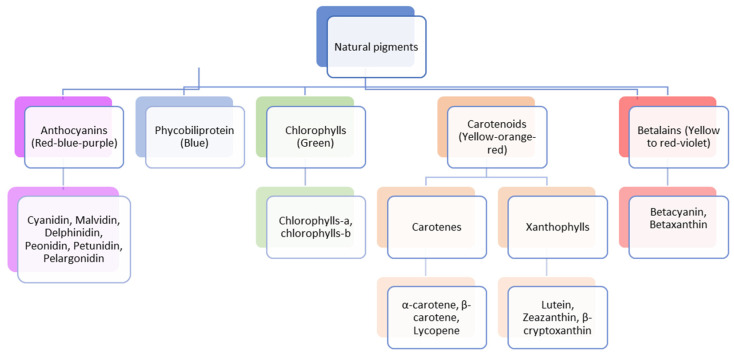
Classification of natural pigments extracted from food by-products.

Natural pigments are classified based on their solubility (water- and/or fat-soluble) and their chemical nature (organic or inorganic). Additionally, they are classified based on their structural affinities, solubility, and natural occurrence. Most often, these pigments are divided into five major groups: anthocyanins, betalains, phycobiliproteins, carotenoids, and chlorophylls [4] (Figure 1). However, there are other notable pigment groups, such as xanthones and quinones. These pigments are distributed in various natural kingdoms, including plantae, fungi, and bacteria [5]. However, our focus in this review is on the five major groups mentioned above.

Due to both their coloring properties and potential positive effects on human health, the recovery of natural pigments from food waste is crucial. Conventional solid–liquid extraction techniques for recovering natural pigments from food by-products are often time-consuming, expensive, and unsustainable [6]. In recent years, novel extraction technologies, such as pulsed electric fields, ultrasound, microwave, and high-pressure-assisted extraction, among others, have gained prominence due to the increased consumer demand for nutritious foods produced using environmentally friendly technologies [6]. The benefits of employing novel technologies for extracting natural pigments from plant products include better isolation, higher selectivity, reduced energy consumption, and low environmental impact [7].

These natural pigments, which can be isolated from food by-products, may possess potential bioactivities, such as antimicrobial, antioxidant, antiproliferative, and anti-inflammatory properties [8]. Due to their significant nutritional and beneficial health properties, these pigments can be considered functional food ingredients, playing a crucial role in food production by masking unpleasant attributes or enhancing the natural properties of food products. As a result of the substantial number of by-products produced daily in the food industry, it is essential to deepen knowledge in this area. In this context, and considering the abovementioned information, this review paper aims to provide a recent update on novel extraction techniques that are utilized for the recovery of natural pigments, including anthocyanins (ANCs), phycobiliproteins (PBPs), betalains, carotenoids, and chlorophylls, from food by-products. Additionally, the present work offers a relevant and comprehensive approach to natural pigment bioactivities, their health benefits, and related food applications.

## 2. Studies Published in the Last Two Decades—An Overview

### 2.1. Research Methodology

Scientific publications on natural pigments published in the past two decades (2003–2023) were obtained through a search on the Web of Science using the keywords “natural pigments”, “food”, and ‘’by-products”, and by selecting the option “all fields”. This approach ensured the inclusion of all papers available in the Web of Science databases and that featured the terms “natural pigments”, “food”, and “by-products” in their title, abstract, or keywords. The abstracts of all papers were analyzed to classify them into one of four categories: extraction technologies, bioactive properties, health benefits, or food applications. Abstracts of meetings, patents, papers not written in English, or where food was not analyzed as a by-product were eliminated.

### 2.2. Results

**Figure 2:**
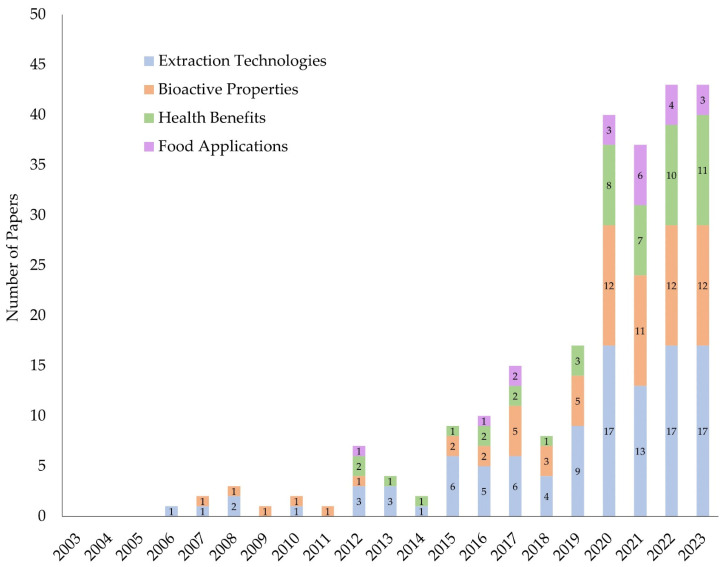
Number of papers from the last two decades that are related to the extraction technologies, bioactive properties, health benefits, and food applications of natural pigments. The papers were analyzed, selected, and divided according to the specified criteria.

Figure 2 shows the number of research papers focusing on natural pigments extracted from food by-products published over the last two decades. According to our search, 245 papers have been published since 2006. The data highlights a significant increasing trend in the number of publications: 7 papers from 2003 to 2009, 75 papers from 2010 to 2019, and notably, a sharp increase to 163 papers from 2020 to 2023. This trend indicates a growing interest and expansion in research within this area of study.

## 3. Natural Pigments

Natural pigments are organic compounds responsible for the vibrant colors found in various fruits, vegetables, flowers, and other living organisms [9]. These pigments play a crucial role in both the aesthetic appeal of natural products and their physiological functions [10].

Anthocyanins are water-soluble pigments found in berries, grapes, red cabbage, and other fruits and vegetables [11]. They are powerful antioxidants that help combat oxidative stress and inflammation in the body, potentially reducing the risk of chronic diseases [12]. Consumption of anthocyanin-rich foods has been linked to improved cardiovascular health, cognitive function, and eye health [13].

Carotenoids, including beta-carotene, lutein, and lycopene, are lipid-soluble pigments found in carrots, tomatoes, sweet potatoes, and leafy greens [14]. These pigments are known for their role in eye health, particularly in preventing age-related macular degeneration [15]. They also support immune function and skin health due to their antioxidant properties [16,17].

Chlorophylls are green pigments found in all green plants, algae, and some bacteria. They are essential for photosynthesis, allowing plants to convert sunlight into energy [18] Chlorophylls have been studied for their potential detoxifying properties and ability to bind to carcinogens, possibly reducing the risk of cancer [18,19].

Betalains are water-soluble pigments found in beets, Swiss chard, and cactus fruits [20]. They exhibit strong antioxidant and anti-inflammatory properties and have been associated with reduced oxidative stress and improved cardiovascular health [21]. Betalains also show potential in supporting liver function and overall detoxification processes [22].

Phycobiliproteins are water-soluble pigments found in cyanobacteria, red algae, and some cryptomonads [23]. They are integral components of the light-harvesting complexes in these organisms, playing a crucial role in photosynthesis [24]. Phycobiliproteins, such as phycocyanin and phycoerythrin, are known for their vibrant blue and red colors, respectively [23]. These pigments have shown potential health benefits, including anti-inflammatory, antioxidant, and anticancer properties [24]. Phycocyanin, in particular, has been studied for its ability to protect against oxidative stress and support immune function [25,26].

Moreover, these pigments may contribute to overall well-being by supporting cellular health and providing essential vitamins and minerals [9,10,14]. Due to their nutritional significance and visual impact, natural pigments, such as anthocyanins, carotenoids, phycobiliproteins, betalains, and chlorophylls, are gaining attention in various industries, including food, cosmetics, pharmacology, and textiles, as alternatives to synthetic dyes [10]. Hence, the increasing consumer demand for natural and sustainable products has fueled the exploration and use of plant-derived pigments [4].

**Figure 3:**
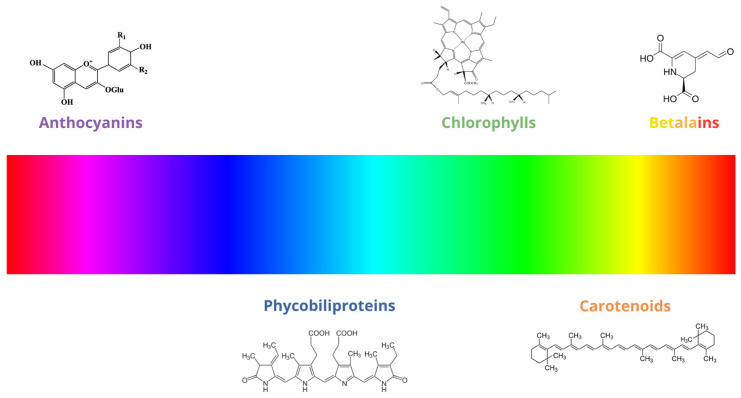
Color spectrum of natural pigments.

Figure 3 depicts several chemical structures from these natural colorants.

### 3.1. Anthocyanins

Anthocyanins, a subgroup of flavonoids, are vibrant plant pigments responsible for the rich red, purple, and blue colors seen in many fruits, vegetables, and flowers [11]. These water-soluble vacuolar polyphenolic pigments, which are synthesized in plants via the phenylpropanoid pathway, encompass six primary forms: pelargonidin, cyanidin, peonidin, delphinidin, petunidin, and malvidin [27,28]. ANCs are also recognized for their significant health attributes, including anti-cancer properties associated with both chemopreventive and chemoprotective effects observed in vivo and in vitro across various cell lines [29], as well as antioxidant and anti-inflammatory benefits [30,31].

The chemical structure of ANCs involves an aglycone, to which the sugar binds through beta (b)-glycosidic interactions, such as glucose, galactose, rhamnose, and arabinose, commonly conjugated to the C3 hydroxyl group in the C ring [32]. Color variations result from the pH-dependent transformation of ANCs, with acidic conditions favoring a reddish hue and alkaline conditions leading to a blue or purple color [32,33]. Their stability is also influenced by other factors, such as pH, temperature, and light exposure [32].

The distinctions between ANCs lie in the number of hydroxyl groups in the molecule, the degree of methylation of these hydroxyl groups, the nature and number of sugars bound to the molecule, their position of binding, and the nature and number of aliphatic or aromatic acids attached to the sugars [34]. They are soluble in water, which facilitates their use as colorants in foods. In addition, some are extracted from grape skins and utilized as natural pigments in beverages and dairy products. Thus, in the winemaking process, it is possible to use the grape pomace to extract ANCs, contributing to zero waste and, consequently, promoting a circular economy [35].

### 3.2. Phycobiliproteins

Phycobiliproteins are water-soluble pigmented proteins found in certain types of photosynthetic organisms, notably in cyanobacteria (blue–green algae) and red algae. They are responsible for the vivid colors observed in these organisms [36]. These pigments, involved in photosynthesis, are produced through the association of fluorescent phycobiliproteins with the thylakoid membrane within the chloroplasts of algae. Chemically, they consist of chromophores (bilins or open-chain tetrapyrroles) connected to an apoprotein through thioether covalent bonds, resulting in varied spectral properties [32].

PBPs are comprised of a protein–pigment complex and are divided into four types: phycoerythrins (red, λmax = 540–570 nm), phycoerythrocyanins (orange, λmax = 560–600 nm), phycocyanins (blue, λmax = 610–620 nm), and allophycocyanins (bluish green, λmax = 650–655 nm) [37], allowing them to capture light across a broad range of wavelengths [26]. Phycobiliproteins represent an alternative origin of blue-colored protein pigments, exhibiting greater stability compared to ANCs at pH levels beyond the blue range for the latter substances (pH: 5–7). It is worth noting that ANCs, on the other hand, display increased stability under acidic pH conditions [38]. Furthermore, PBPs are particularly thermolabile, reducing their color intensity after 30 min in neutral solutions at 60 °C [32]. As a substitute for employing these pigments, technological procedures, such as high-pressure processing, have been utilized to pasteurize drinks at low temperatures [39].

PBPs have demonstrated diverse biological activities, mainly attributed to their chromophore component, showcasing their potential applications across various biotechnological fields [40]. Their antioxidant properties are particularly significant, as these proteins help neutralize free radicals in the body, potentially reducing oxidative stress and preventing cellular damage. Additionally, phycobiliproteins exhibit antibacterial characteristics, suggesting potential roles in supporting the immune system against microbial infections. Studies have also highlighted their antitumor properties, indicating potential applications in cancer prevention and therapy. The diverse range of health benefits associated with phycobiliproteins makes them compelling candidates for incorporation into nutraceuticals and dietary supplements, contributing to overall well-being and offering promising avenues for health-focused research and applications [24,41,42,43,44].

### 3.3. Chlorophylls

Chlorophylls are green pigments found in the chloroplasts of plants, algae, and cyanobacteria, playing a central role in photosynthesis [45]. Structurally, chlorophylls possess a porphyrinic structure, consisting of four pyrrole rings (C_4_H_4_NH) arranged around a central magnesium ion. This magnesium ion is coordinated within the structure, and an elongated hydrophobic alkyl chain is linked to it [46]. The chlorophyll molecules, featuring a hydrophilic porphyrin group head and a lipophilic hydrocarbon tail known as the phytol group, are generally considered insoluble in polar solvents due to their lipophilic hydrocarbon chains [8].

Chlorophylls exhibit two structures in plants, where chlorophyll-a (C_55_H_72_MgO_5_N_4_) features a methyl (–CH_3_) group at the carbon-7 position, and chlorophyll-b (C_55_H_70_MgN_4_O_6_) has an aldehyde (–CHO) group at the same position. These structural differences lead to variations in color, with chlorophyll-a appearing as blue–green and chlorophyll-b as blue–yellow in a ratio of 3:1 in chloroplasts, respectively [6]. Depending on the temperature, oxygen availability, and pH variations, chlorophylls can form a variety of derivatives (pheophytins, chlorophyllides, phephorbides, piroderivatives, chlorin-type derivatives, and other allomerized compounds) [47].

The bioactive properties of chlorophylls are attributed to their ability to function as antioxidants, antimutagens, and anticarcinogens. Their distinctive chemical structure allows chlorophylls to effectively neutralize harmful free radicals, alleviate DNA damage, and regulate cellular processes implicated in the onset of diseases. Additionally, their hydrophobic side chains facilitate interactions with biological membranes, influencing cellular uptake and impacting signaling pathways [48].

### 3.4. Carotenoids

Carotenoids constitute a diverse group of naturally occurring pigments found in various autotrophs, such as microalgae, bacteria, fungi, and plants [15]. These lipophilic compounds [17] belong to the tetraterpene family, characterized by a central chain with 40 atoms of carbon, varying the alternation of single and double bonds according to the specific carotenoid [15]. These natural pigments can be classified based on their chemical composition as (i) carotenes, containing only carbon and hydrogen (e.g., alpha (α)-carotene, β-carotene, and lycopene), or (ii) xanthophylls, containing oxygenated derivatives at the cyclic end groups (e.g., β-cryptoxanthin, lutein, and zeaxanthin) [49,50].

Carotenoids, with their vibrant colors in fruits and vegetables [51], offer a spectrum of health benefits. As antioxidants, they play a crucial role in neutralizing harmful free radicals, contributing to cellular health, and potentially reducing the risk of chronic diseases [15,17,52,53]. While β-carotene, a precursor to vitamin A, supports healthy vision, skin, and immune function [17,54,55] lutein and zeaxanthin are associated with protection against age-related macular degeneration [17,54,56,57]. Lycopene, found in tomatoes and watermelon, has been linked to a lower risk of certain cancers [17,54].

However, the reduced bioaccessibility of carotenoids [55] depends on certain factors, like the food matrix and processing methods [52,58,59]. While some carotenoids are readily absorbed, others may benefit from cooking or processing that breaks down cell walls, enhancing their release and absorption [59]. Since humans cannot synthesize carotenoids themselves, the consumption of a variety of colorful fruits and vegetables in the diet ensures a diverse intake of these compounds, maximizing their bioavailability and supporting overall health [60,61].

### 3.5. Betalains

Betalains, vacuolar nitrogen-containing pigments found in approximately 17 plant families within the *Caryophyllales* order, exhibit a fascinating diversity of colors [62]. Categorized into two subclasses, betacyanins manifest as red to violet hues, while betaxanthins contribute yellow to orange tones [63]. Remarkably, despite their seemingly similar functions, betalains and ANCs, another group of plant pigments, have never been co-located in the same plant, suggesting a mutual exclusivity between them [64].

Accumulating in the vacuoles of cells, particularly in the epidermal and subepidermal tissues of betalain-synthesizing plants, these hydrophilic pigments impart vibrant colors to flowers in various genera, such as *Mirabilis*, *Glottiphylum*, and *Portulaca* [64]. In contrast, within *Caryophyllales*, ANCs dictate coloration in some families, like *Caryophylaceae* and *Molluginaceae*. The distinctive distribution and characteristics of betalains underscore their unique role in contributing to the chromatic diversity observed in flowering plants [63].

Betalains are also recognized for their potential health benefits. As potent antioxidants, betalains contribute to cellular health by neutralizing free radicals, which are associated with various chronic diseases [65]. Additionally, the anti-inflammatory properties attributed to betalains may help mitigate the risk of inflammatory-related conditions [66]. Some studies suggest potential cardiovascular benefits, with betalains supporting heart health by improving blood vessel function and reducing oxidative stress [22]. While ongoing research continues to explore the full extent of these health advantages, incorporating betalain-rich foods into a balanced diet, such as colorful vegetables, stands as a potential means to promote overall well-being [22].

These pigments have been used in food systems in a variety of ways, thanks to technological advances. However, because these pigments are light- and heat-sensitive, they may impart an unpleasant earthy flavor to food preparations. Nonetheless, betalains outperform ANCs in terms of water solubility, color potential, and neutral pH stability [32].

## 4. Extraction Methodologies for Natural Pigments Recovery

The most common method used to obtain pigments from their natural sources is extraction through solvents. The most popular solvents applied for this purpose are pure methanol, isopropanol, and ethanol, as well as aqueous mixes [67,68,69,70,71] Despite this, ethyl acetate, hexane, acetone, and dimethyl sulfoxide (DMSO) are also used [72,73,74]. Together with solvent extraction, enzymes can also be utilized to achieve higher yields of extraction [75]. Furthermore, many techniques can be used to assist and ensure an effective extraction of various pigments [71,76,77,78,79,80,81,82,83,84,85]. In this way, several methodologies employed for pigment extraction are described below, while extraction conditions and application examples are detailed in Table 1.

### 4.1. Ultrasound-Assisted Extraction

The extraction with an ultrasound system is accomplished by a process known as ultrasonic acoustic cavitation, in which microscopic bubbles are formed in the collapsing cells. This leads to cells shattering, which facilitates the release of bioactive substances. There is an increase in the number of phytoconstituents, such as pigments, that can be extracted thanks to the exudation of the potential components of the shattered cell [86] For instance, Luengo et al. [82] extracted successfully carotenes from dry tomato pomace, using an ultrasound-assisted extraction, applying a low sample to solvent (25% *v*/*v* hexane to ethanol) ratio (3% *w*/*v*), at 25 °C) and at a relatively short extraction time (10 min manosonication), obtaining higher yields (8.43 ± 0.26% *w*/*w*) when compared with the control (3.54 ± 0.25%), without ultrasound (Table 1). Therefore, the advantages of this method include the speed of extraction, the reduced use of solvents, and the low energy consumption. The main disadvantage of this methodology is related to the degradation of thermolabile compounds due to the high temperatures used. This happens due to the heating of water molecules contained in the sample. This warmth causes evaporation, which puts enormous strain on the cell wall, weakening it from the inside until it eventually breaks [77].

### 4.2. Microwave-Assisted Extraction

Microwave-assisted extraction is a viable method for acquiring natural pigments. This technique enables the simultaneous extraction of multiple samples, can be conducted alongside agitation processes, consumes lower energy and solvent quantities, and eliminates the need for sample dehydration or preparation [87]. Additionally, it offers flexibility in terms of sample sizing. For instance, this method has been reported in the literature, combined with ultrasound-assisted extraction, for the extraction of lycopene (yield of 97.4%, *v*/*v*) from non-compliant tomatoes [83] (Table 1). Nonetheless, some of the limitations related to this technique include restrictions on the solvents used, which can impact the method’s selectivity. Additionally, the consequent extraction residues may require a subsequent separation method, and the elevated temperatures involved may contribute to the degradation of thermolabile compounds [85].

### 4.3. Supercritical Fluid Extraction

Bioactive compounds, such as natural pigments, can also be obtained from a diverse matrix through supercritical fluid extraction. In most cases, carbon dioxide is employed as the solvent throughout the extraction process. This is achieved by operating at pressure and temperature levels higher than the critical point of the component being extracted or the mixture that contains it. When the pressure is released, the supercritical fluid can be easily separated from the compound of interest and leaves next to no residues, providing extracts of considerable high purity. Moreover, this procedure usually takes short amounts of time, and the applied fluid is typically non-toxic [88] Pan-Utai et al. [89] used the CO_2_ supercritical fluid method to extract chlorophyll, carotenoids, and phycobiliproteins from *Arthrospira platensis* biomass, rendering 133.73 mg/mg dry weight (DW), 77.95 µg/mg DW, and 73.40 mg/g DW, respectively (Table 1). However, the downsides of this methodology are related to its operating costs and the availability of the equipment. Moreover, due to the high pressures involved in the process, it is challenging to continually add solids to the extract. In addition, when co-solvents are used to change the polarity of a fluid, they need to be removed through further extract purification, leading to extra steps.

### 4.4. Subcritical Water Extraction

Subcritical water extraction operates based on water’s inherent properties, namely structural and thermodynamic ones. The dielectric constant of water lowers in the range of organic solvents when subjected to high temperature and pressure and, as a result, water can dissolve analytes with polarities ranging from low to medium when these conditions are met. The extraction of biocompounds is accomplished by the mechanisms of mass transfer, which include diffusion and convection. The solute–matrix and solute–solute interactions are disrupted by the high temperature present throughout the operation. This results in a decrease in the activation energy required for the desorption procedure. Furthermore, the use of high pressure is beneficial to the extraction process, as it compels the water to penetrate deeper into the matrix pores [90]. Regarding the acquisition of natural pigments, this technique has also been used for the extraction of chlorophyll A and B (yields of 1.58 ± 0.41 mg/g and 1.90 ± 0.05 mg/g DW, respectively), and carotenoids (yield of 3.62 ± 0.08 mg/100 g DW) from *Stevia rebaudiana bertoni* leaves [80] (Table 1). Despite the high costs of this method and limitations regarding by-product volume, due to the high pressures involved, pigment extraction by the subcritical water process can be useful, since it is relatively fast to carry out and allows an increased solvent diffusion [91].

### 4.5. Pressurized Fluid Extraction

In order to accomplish extraction by pressurized fluids, the solvent and the matrix that contains the target bioactive compounds are placed inside an extraction cell, where the solvent is brought to a condition identical to its super-critical state. Therefore, the high temperatures and pressures applied enhance the solubility of analytes, promoting increased diffusion within the sample, while the pressure applied raises the solvent’s boiling point. During this process, the surface tension and viscosity of the solvent drop simultaneously, increasing the analyte’s ability to be dissolved in the solvent, which leads to a higher rate of mass transfer. Moreover, these conditions allow a faster extraction process and reduce the quantity of solvent needed [92] Considering these advantages, Saldaña et al. [78] extracted ANCs from cranberry pomace using the pressurized ethanol, leading to a yield of 7.78 mg of cyanidin-3-glucoside equivalents. Moreover, this research concluded that the solvent choice could influence the yield of extraction, since the usage of ethanol resulted in higher ANCs amounts compared to the use of water by itself (yield of around 1.00 mg cyanidin-3-glucoside equivalents) at the same conditions (Table 1).

While the practical application of pressurized fluids methodology holds promise for natural pigment extraction, it is noteworthy to mention that the high temperatures required for this method may lead to an increase in the solubility of various molecules, in addition to the target compounds. Thus, pressurized fluids extraction may be less selective than other techniques, and certain chemical reactions are more likely to occur due to the elevated temperatures, potentially resulting in the formation of hazardous chemicals [93].

### 4.6. Pulsed Electric Fields

The usage of pulsed electric fields for the extraction of natural compounds is based on the production of a potential difference between a conductive biological material placed between two electrodes. Also, variables, such as the voltage, shape, and distance between the electrodes, as well as medium conductivity, will affect the produced electric field which, consequently, will act on the cellular organelles’ membrane [94] A practical example of the application of this technique regarding pigment extraction was reported by Nowacka et al. [76], who obtained improved yields of betalains from beetroot after using pulsed electric fields (betanin: 4.40 mg/100 g DW; vulgaxanthin: 3.20 mg/100 g DW) (Table 1). Added advantages to this method are related to the low level of compound degradation and high degree of selectivity. However, a drawback lies in the substantial cost associated with the equipment, which can limit the accessibility and incorporation of this technology [95].

### 4.7. Enzyme-Assisted Extraction (EAE)

Enzyme-assisted extraction (EAE) of pigments relies on enzymatic hydrolysis to break down plant cell walls. The effectiveness of EAE depends on various factors, including enzyme type and dosage, process time and temperature, plant material properties (particle size, water content, and chemical composition), and the solvent-to-solid ratio [96].

In EAE, plant material is pretreated with protease, pectinase, pectin esterase, cellulase, hemicellulase, cellobiase, α-amylase, and fucosyltransferase. These enzymes hydrolyze the cell walls, releasing phytochemicals bound to lipid and carbohydrate chains. Following this pretreatment, extraction is performed by solvent (e.g., water) extraction or pressurized hot water extraction to obtain volatile compounds, hydrophilic and hydrophobic pigments, phenolic compounds, and other bioactive substances from the plant samples [97]. Furthermore, EAE can be considered an environmentally friendly process, due to the use of water and natural enzymes, and offers several advantages, including higher extraction yields, improved extract quality, scalability, and the reduced need for subsequent filtration and purification steps [97].

This method has successfully recovered various compounds from plant tissues, including carotenoids, lycopene, betalains, and chlorophylls (Table 1). Strati et al. have recovered carotenoids and lycopene from tomato waste using this technique. The highest extraction yields of total carotenoids (127 mg/kg DW) and lycopene (89.4 mg/kg DW) were achieved in enzyme-treated samples using ethyl lactate as the solvent, (10:1 *v*/*w*) [98]. Mazzocchi et al. conducted a design experiment (DOE) to determine the optimal process parameters for chlorophyll extraction. The parameters studied included temperature (°C), time (hours), enzyme dose (U/g), ZnCl₂ dose (ppm), and buffer/substrate ratio (B/S). Using a response surface methodology, they identified the conditions that maximized chlorophyll yield and ensured the best green quality. The optimal conditions were a temperature of 25 °C, ZnCl₂ concentration of 150 ppm, B/S ratio of 17.5, less than 2 h processing time, and an enzyme dose between 12 and 45 U/g. Additionally, the study demonstrated that the chlorophyll-based extract could effectively impart various green colorations to real food products [99].

**Table 1:** Examples of methodologies, extraction conditions, and respective yields, employed for the acquirement of natural pigments from different sources.

By-Product	Pigment	Extraction Method	Solvents/ Co-Solvents	Other Conditions	Yield	References
Cantaloupes peels	Carotenoids	Ultrasound-assisted extraction	Optimal solvent: hexane/ethanol (1:1, *v*/*v*).	3.3% (*w*/*v*) sample to solvent ratio; 21.0 ± 2.0 °C.	Yield obtained when working under optimal conditions: 107.74 µg βCE/g DW of by-product.	[71]
Tomato pomace	Carotenoids	Ultrasound-assisted extraction	Hexane/ethanol (1:4, *v*/*v*)	3% (*w*/*v*) sample to solvent ratio; 25 °C; 10 min.	8.43 ± 0.26% (*w*/*w*)	[82]
Tomato peels	Carotenoids	Solvent extraction	Ethanol/acetone/hexane (3:1:1, *v*/*v*/*v*)	13.3% (*w*/*v*) sample to solvent ratio; 40 °C; 23.5 min.	128 mg βCE/100 g DW of by-product.	[72]
*Opuntia* spp. peels	Betacyanins	Solvent extraction	Ethanol/water (80:20 *v*/*v*)	4% (*w*/*v*) sample to solvent ratio; 25 °C; 150 rpm agitation; 1 h.	Extraction from *Opuntia ficus-indica vargialla:* 1.25 ± 0.01 mg/g DW of extract; extraction from *Opuntia ficus-indica* var. *sanguigna:* 3.97 ± 0.03 mg/g DW of extract; extraction from *Opuntia engelmannii:* 19.4 ± 0.4 mg/g DW of extract.	[74]
Raspberries pomace	Anthocyanins	Enzymatic extraction	100% distilled water (*v*/*v*)	Enzymes tested: polygalacturonase, pectinmethylesterase, or polygalacturonase + cellulase; enzyme dosage of 10 mL/100 kg of by-product; 45 °C; 1 h.	Extraction with polygalacturonase: 0.25 ± 0.018 mg/g FW of by-product; extraction with pectinmethylesterase: 0.24 ± 0.017 mg/g FW of by-product; extraction with polygalacturonase + cellulase: 0.32 ± 0.022 mg/g FW of by-product.	[100]
Non-compliant tomatoes	Lycopene	Ultrasound- and Microwave-assisted extraction	100% ethyl acetate (*v*/*v*)	Optimal conditions: 10.6:1 (*w*/*v*) sample to solvent ratio; microwave power, 98 W; ultrasound power, 50 W; 6.2 min.	Yields obtained when working under optimal conditions: 97.4% (*v*/*v*).	[83,87]
Carrot and spinach	β-carotene	Microwave-assisted and in vacuo extraction	Acetone/ethanol (1:2 *v*/*v*)	8.3% (*w*/*v*) sample to solvent ratio; 25 °C; 20 min; degree of vacuum at 0.04 Mpa.	9.86 mg/100 g FW of carrot; 0.6 mg/100 g FW of spinach.	[85,87]
*Hylocereus polyrhizus* peels	Betaines	CO_2_ supercritical fluid extraction	50% ethanol (*v*/*v*)	1:10 (*w*/*v*) sample to ethanol ratio (pre-extraction); 40 °C; 30 Mpa.	Four fractions were obtained (F1, F2, F3, F4): F1: 30.67 mg/100 g DW of by-product; F2: 15.84 mg/100 g DW of by-product, F3: 12.80 mg/100 g DW of by-product; F4: 29.92 mg/100 g DW of by-product.	[81,101]
*Arthrospira platensis* biomass	Chlorophyll; carotenoids and phycobiliproteins	CO_2_ supercritical fluid extraction	Optimal solvents: distilled water; phycobiliproteins: phosphate buffer	Optimal conditions: 350 bar; 50 °C (for chlorophylls and carotenoids extraction)/40 °C (for phycobiliproteins extraction); static extraction for 60 min and dynamic extraction for 240 min; flow rate of 3 L/min.	Chlorophyll: 133.73 mg/mg DW of biomass; carotenoids: 77.95 µg/mg DW of biomass; phycobiliprotein: 73.40 mg/g DW of biomass.	[89,90]
*Stevia rebaudiana Bertoni* leaves	Chlorophyll A; chlorophyll B and carotenoids	Subcritical water extraction	100% distilled water (*v*/*v*)	Optimal extraction conditions: 60 min of sonication (40 kHz); constant pressure of 10.34 Mpa; mixed with diatomaceous earth (1:3 *w*/*v*); 60% (*v*/*w*) solvent flushing volume; 160 °C; 10 min.	Yields (optimal conditions): chlorophyll A: 1.58 ± 0.41 mg/g DW of by-product; chlorophyll B: 1.90 ± 0.05 mg/g DW of by-product; carotenoids: 3.62 ± 0.08 mg/100 g DW of by-product.	[80,90]
*Arthropira platensis* biomass	Phycobiliproteins	Pressurized fluid extraction	0.1 mol/L sodium phosphate buffer, pH 7	Optimal conditions: 10% (*w*/*v*) sample to solvent ratio; 100 bar; 360 min.	Yield (optimal conditions): phycocyanin: 4.4 g/L of extract; allophycocyanin: 1.6 g/L of extract.	[79]
Cranberry pomace	Anthocyanins	Pressurized fluid extraction	Optimal solvent: 100% ethanol (*v*/*v*)	Optimal conditions: 10% (*w*/*v*) sample to solvent ratio; 25 g of 3 mm glass beads; flow rate of 5 mL/min; 50 bar; 60–120 °C; 10 min.	Yield (optimal conditions): 7.78 mg of cyanidin-3-glucoside equivalent.	[78]
*Rhodotorula glutinis* biomass	Carotenoids	Pulsed electric field-assisted	96% ethanol (*v*/*v*)	Flow rate of 4 L/h; residence time of 1.09 s; 15 kV/cm; 150 µs; 10 °C–40 °C; 1 h of incubation, using a ratio of 1:1 (*v*/*v*) suspension to 96% ethanol (*v*/*v*).	Yield (optimal conditions): 240 μg/g DW of biomass	[77]
Beetroot	Betanin and vulgaxanthin	Pulsed electric field-assisted	Phosphate buffer, pH 6.5	1% (*v*/*v*) sample to solvent ratio; 4.38 kV/cm; pulse number of 20; 4.10 kJ/kg; 20 min of solvent incubation.	Yield (optimal conditions): 4.4 mg betanin/100 g DW of by-product; around 3.2 mg vulgaxanthin/100 g DW of by-product.	[76]
Sunflower waste	Carotenoids	Enzyme-assisted extraction	Hexane, limonene; sunflower oil, turpentine; menthol; M/Hac; M/HLaur; M/HLac (d,l-menthol:d,l-lactic acid)	Multi-enzyme complex viscozyme; optimal conditions: solvent/water: 0.6; sunflower/liquid: 0.015; enzyme: 0.5%.	Yield (optimal conditions): 1449 mg/100 g biomass.	[102]
Tomato waste	Carotenoids/ lycopene	Enzyme-assisted extraction	Hexane, acetone, and ethyl lactate	Incubation temperature: pectinase (45 °C) and cellulase (55 °C); optimal conditions: enzyme-treated samples with ethyl lactate (solvent–solid = 10:1 mL:g).	Pectinase increased the extraction of total carotenoids/lycopene, compared to cellulase. Yield (optimal conditions): total carotenoid (127 mg/kg DW) and lycopene (89.4 mg/kg DW).	[98]
Unsold red beets	Betalains: betacyanins and betaxanthins	Enzyme-assisted extraction	Acetate buffer	Tailored enzymatic mix: Cellulase (37%), xylanase (35%), and pectinase (28%); optimal conditions: 25 U/g total dose of enzymatic mix, temperature 25 °C, and processing time 240 min.	Yield (optimal conditions): (betaxanthins = 11.37 ± 0.45 and Betacyanins = 14.67 ± 0.49 (mg/L)/U).	[103]
Spinach pulp	Chlorophyll	Enzyme-assisted extraction	Ethanol	Pectinex Ultra SP-L; optimal conditions: 8% enzyme concentration, 45 °C, and 30 min.	Yield (optimal conditions): 50.747 mg of total chlorophyll content/100 g spinach pulp.	[104]
Spinach leaves	Chlorophyll	Enzyme-assisted extraction	McIlvaine buffer (pH 5; 0.1 M)	Optimal conditions (highest amount of recovered chlorophyll, and best quality of green (T: 25 °C, Zn: 150 ppm e B/S: 17.5, t: <2 h, and enzyme mix dose between 12 and 45 U/g).	Yield (optimal conditions/design of experiment (DOE)): 1299 µg/g.	[99]

βCE: β-carotenoids equivalents; DW: dry weight; FW: fresh weight.

## 5. Bioactivities of Pigments Recovered from Food By-Products

Natural pigments isolated from waste and by-products exhibit a wide range of bioactivities, presenting promising pathways for diverse applications. These pigments possess inherent antioxidant properties due to their high content of phytochemical compounds, playing a crucial role in scavenging free radicals in the body, thereby reducing oxidative stress, and potentially mitigating the risk of chronic diseases, such as cancer, cardiovascular disorders, and neurodegenerative conditions [105]. Additionally, these pigments have demonstrated anti-inflammatory effects, which can contribute to alleviating inflammatory conditions within the body, including arthritis and inflammatory bowel diseases [105]. Furthermore, some pigments have been showing antimicrobial properties, inhibiting the growth of pathogenic bacteria and fungi. This feature may be beneficial for food preservation and combating microbial infections [106]. Moreover, research suggests that certain pigments may possess anti-obesity properties by modulating lipid metabolism and adipocyte differentiation, presenting an innovative approach to addressing obesity and its associated health risks [107]. Due to their significant nutritional and beneficial health properties as well as phyto-pigment-antioxidant nature, these plants’ secondary metabolites can be considered functional food ingredients [8].

Overall, in recent years, the bioactivities of pigments recovered from food by-products, such as ANCs, phycobiliproteins, chlorophylls, carotenoids, and betalains, have been explored for both human health and innovation in the food industry (Table 2). For instance, Coelho et al. [84] reported that grape by-products rich in ANCs exhibited enhanced antioxidant activity (ORAC assay: 2.02–2.34 g/100 g ascorbic acid equivalent) and antimicrobial potential against *Yersinia enterocolitica*, *Pseudomonas aeruginosa*, *Salmonella enteritidis*, *Staphylococcus aureus*, and *Bacillus cereus.* Szymanowsk et al. [108] showed that ANCs present in raspberry pomace confer anti-inflammatory activity, particularly COX-2 inhibitory properties, with the highest inhibitory activity at IC_50_ = 0.87 mg FW/mL. Phycobiliproteins recovered from *Spirulina sp.* showed antioxidant and anti-inflammatory activity [109]. Moreover, chlorophylls and carotenoids have been reported in numerous studies proving that these two classes of pigments hold promising antioxidant activity [50]. Regarding betalains presented in red dragon fruit peels, Rodriguez et al. [110] reported different bioactivities, namely antioxidant, anti-inflammatory, and antigenic.

The reported bioactivities in Table 2 highlight that pigments obtained from food wastes and by-products can exhibit strong bioactivities. Nonetheless, future research is essential to explore the biological potential of these pigments for their utilization in the food industry.

## 6. Application of Pigments in the Food Industry and Potential Health Benefits

Artificial food colorants have been raising some concerns due to the formation of toxic compounds upon their use in food production as additives. They have also been associated with poor food technology practices, leading to discussions about food safety [124]. Furthermore, data suggest that certain artificial pigments are linked to allergic responses, the development of some forms of cancer, and even attention deficit in younger people [125].

Regarding the legislation governing the application of these compounds in foods, the Food and Drug Administration (FDA) and European Food Safety Authority (EFSA) agencies control the use of colorants as food additives through standards of practice, involving thorough tests to evaluate their toxicity before permitting their inclusion in the market. However, American legislation does not distinguish pigments derived from natural sources and those manufactured through synthesis [126,127]. Over the past fifty years, there has been a drastic increase in the proportion of foods containing synthetic colorants [128], such as azo dyes (chromophores). The metabolism of these substances results in the production of free aromatic amines in the gut lumen [129], some of which have the potential to cause cancer and mutations [130,131]. Recent research has established a connection between the use of the artificial colorant Allura Red AC (AR) and an elevation in 5-hydroxytryptamine (5-HT), indicating an increased risk of developing colitis [129]. Even though there are some studies available on artificial coloring agents, there is still a need for additional toxicological research concerning natural pigments.

In the European Union, natural pigments must be extracted from plant sources before being used to color the final food product. The stability of extracted pigments is affected by environmental and chemical conditions during processing or storage, such as pH, exposure to light, and the presence of metal ions, oxygen, and enzymes; however, high temperatures have the greatest impact [132]. Consequently, introducing natural pigments into food systems requires efficient ways to increase their stability throughout processing and storage. Considering this issue, the need for viable alternatives to artificial food colorants has emerged.

Natural pigments have been researched for their diverse applications in the food industry. Moreover, they can also possess nutritional properties and beneficial pharmacological effects. Therefore, interest in using natural pigments in the food industry has been sparked by their overall health improvement capacities, serving as viable replacements for artificial food colorants [26,124]. In this way, among the several classes of natural pigments, each can be particularly employed depending on the intended application, given their distinct physicochemical and functional characteristics.

Nevertheless, natural pigments face several limitations, including low bioavailability, stability issues, and potential mild health impacts, which restrict their applications in food. However, encapsulation techniques have effectively addressed these challenges by protecting the pigments from degradation. Encapsulation not only enhances the stability of natural pigments but also improves their bioavailability and health-promoting activities [133].

### 6.1. Anthocyanins

ANCs are pigments found in a wide variety of fruits and vegetables (e.g., purple berries, grapes, plums), presenting a purplish-red color. Due to their attractive color and potential bioactivities [84,111,112], these pigments can be recovered from by-products of those foods and applied in various products, such as drinks, sweets, ice cream, and other dairy goods [33,35,134].

For example, cupcakes developed using ANCs derived from Roselle (*Hibiscus sabdariffa* L.) showed lower total carbs (41.2%), lipids (7.1%), and ash (4.2%) than the control, while preserving 77.00% of the overall ANC content. Moreover, the sensory assessment of color, appearance, texture, taste, volume, and aroma showed no statistically significant variances compared to the control cupcakes [135]. In another study, carried out by Silva et al. [136], the heat-assisted method was used to obtain an ANC-rich extract (cyanidin-*O*-di-hexoside, C3G, Pr3G, cyanidin-3-*O*-xyloside, and cyanidin-3-*O*-dioxayl-glucoside) to produce doughnuts, resulting in lower carbohydrate (59.5 g/100 g FW) and energy levels (307.2 kcal/100 g FW), higher free sugars values (19.3 g/100 g FW), and no significant variations in free fatty acid content, compared to control doughnuts (without ANC-rich extract). Another example of a food application of ANC-rich extracts was brought up by Albuquerque et al. [137] who obtained a heat- and ultrasound-assisted ANC-rich extract from jabuticaba (*Myrciaria jaboticaba* (Vell.) Berg) epicarp to be utilized as a natural colorant in the production of French macarons, showcasing potential applications for co-pigmentation in drinks to stabilize highly degradable pigments [135,136,137].

ANCs have also been combined with other compounds, such as β-cyclodextrin, alginate, and zinc ions, to enhance their stability and prevent degradation, especially in beverages [138,139]. For instance, a study showed that high degradation rates were obtained for anthocyanin powders and coat extracts (9.51–119.93 days) when incorporated in beverages; however, their encapsulation with β-cyclodextrin delayed their degradation (up to 43 months) [140]. In a distinct assessment, the addition of alginate and zinc ions has also been reported to protect ANCs from degradation, upon their addition also on beverages [32].

In addition, ANCs can also act as adjuvants to retain the color of food products during thermal processing. Thus, natural pigments are commonly used in yoghurt production owing to their heat stability during manufacturing and may be simply labelled as “vegetable color”. The most common colorants used for this application are carmine from the cochineal beetle (brilliant red color ranging from “strawberry” to “blackcurrant”), and ANC-rich extracts from mulberry (*Morus rubra*), among others [141]. Benchikh et al. [142] used anthocyanin extracted from strawberries (*Fragaria ananassa*) to enhance the color and antioxidant properties of yoghurt, demonstrating that the incorporation of strawberry ANCs not only improved the visual appeal but also enriched the yoghurt with health-benefiting compounds. Montibeller, M.J et al. [75] produced an ANC-rich extract from grape skin (*Cabernet Sauvignon*) and was incorporated into kefir, and showed decreased pH, increased acidity, and low total soluble solids. Furthermore, the kefir with the addition of anthocyanins as a natural colorant had physical properties similar to natural kefir without additives.

Regarding potential health benefits, peonidin-3-glucoside and delphiniedin-*O*-p-coumarylglugoside ANCs have been shown to inhibit the initiation, promotion, and progression of several cancers, including cervical cancer [143,144], breast cancer [144,145,146,147], lung cancer [148], liver cancer [69,149,150,151], prostate cancer [152], colorectal and intestinal cancers [127,153,154,155,156], fibro-sarcoma [157], metastatic melanoma [158], and blood cancer [159], due to their chemoprotective effect. In addition, ANCs have been the subject of extensive research to determine the molecular mechanisms underlying this kind of activity. These studies suggest that ANCs can inhibit multiple signaling pathways involved in the growth of tumors and the process of apoptosis. In an early study, ANC-rich extract from *Aronia meloncarpa* caused an arrest in the G1/G0 and G2/M phases of the cell cycle in HT-29 colon cancer cells. This was accomplished by increasing the expression of p21WAF1 and p27KIP1 while simultaneously decreasing the expression of cyclin A and cyclin B [159].

### 6.2. Phycobiliproteins

In the food industry, the blue color presents one of the greatest obstacles for producers. In contrast to the readily accessible natural red, yellow, and orange pigments, the natural sources of blue pigment are extremely restricted [86,160]. Nonetheless, consumers may find blue foods more enticing due to their distinct color. Therefore, some blue candies and beverages are often still dyed with artificial colors by food manufacturers. Consequently, when customers see blue-colored food products, they immediately perceive them as highly processed foods and may assume that they hold less nutritional value. However, a class of natural pigments that can be used as an alternative is phycobiliproteins. These compounds can be extracted, for instance, from spirulina (*Arthrospira platensis*) and are predominantly used for color confections, gum, dairy products, and soft drinks [161]. Currently, PBPs are FDA-approved for usage in candies. Additionally, food firms in Belgium, India, and France, among other countries, have already been employing PBPs as beverage components [88]. *Porphyridium*, a red microalgae genus, is a source of fluorescent phycobiliproteins with pigment characteristics particularly interesting for confections. This red or pink colorant can be applied on clear sugar-based candies or cake decorations, as a natural alternative to artificial colorants [162]. Furthermore, regarding the physicochemical characteristics of these compounds, according to Spence et al. [161], PBPs are stable at pH 5.0–7.5 (25 °C) and are reported to lose color intensity when exposed to temperatures around 60 °C, for 30 min, in neutral solutions [161]. Therefore, technological procedures, such as high-pressure processing, have been employed to pasteurize drinks at low temperatures, as a way to preserve these thermolabile compounds [92].

As already mentioned, PBPs are reported to hold several bioactivities which can also be relevant for health applications. PBPs can be utilized as effective anticancer agents due to their free radical scavenging action and can counteract the damage provoked by toxic metals (e.g., chromium) due to their lipid peroxidase activity [40]. Moreover, due to their antioxidant activity, they can be utilized to prevent oxidative stress produced by HgCl_2_, a consequence of acute renal impairment [94]. Furthermore, PBPs can promote cell growth and have neuroprotective effects due to their immunomodulatory functions [163], and they are reported to be able to slow the progression of Alzheimer’s disease by inhibiting β-secretase proteins [164]. Also, PBPs were reported to mitigate radiation-induced acute liver oxidative damage [165]. In addition, as a rich source of protein and critical amino acids, PBPs are overall interesting candidates for nutritional supplements and natural food additives in the food sector [88,92,166].

### 6.3. Chlorophylls

Chlorophylls, the green pigments found in plants and algae, have numerous physiological functions in these organisms and have been also gaining attention for their potential health benefits and diverse food applications [18,167]. Algae biomass, a rich source of chlorophylls, can be added to food products to obtain a functional food with enhanced health benefits, such as the addition of microencapsulated *spirulina* to pasta [168] or microalgae biomass to cookies due to an increase in their bioactive compounds [168]. Chlorophylls are designated as E140i colorants in the food sector, and in the USA these natural pigments are approved for use in the food industry, for example, *Arthrospira platensis* (a source of chlorophyll) in confections, frostings, ice cream, frozen desserts, beverage mixtures, powders, yogurts, custards, and puddings, among other food applications [75].

Chlorophyll can also serve as a natural green food coloring agent, replacing synthetic colorants, to enhance the visual appeal of foods as well as their sensory attributes and nutritional properties by adding chlorophyll extract obtained from seaweed in a jelly dessert [45]. However, due to the instability of chlorophylls in different temperatures, food components, light, oxygen, pH, packaging materials, and storage conditions, it could be difficult to use them as coloring additives [169]. A chlorophyll nano-emulsion from pomelo leaves was created to increase the stability of chlorophylls being demonstrated that could be used for its health-beneficial effect due to the observed high stability [170]. Nevertheless, additional investigation is necessary to improve the stability of natural color additives. Additionally, chlorophylls have been associated with a variety of health advantages as nutraceutical agents due to their antioxidant, antimutagenic, and anti-inflammatory properties [171,172,173].

Numerous studies have evaluated the efficacy of chlorophylls and chlorophyll derivatives as antiproliferative and pro-apoptotic agents in a variety of cancer cell lines and animal models [174]. Cheng et al. [175] stated that three pheophorbide compounds, which are produced by the breakdown of chlorophyll isolated from the leaves and stems of *Clerodendrum calamitosum*, exhibited strong cytotoxicity against human lung carcinoma (A549), ileocecal carcinoma (HCT-8), kidney carcinoma (CAKI-1), breast adenocarcinoma (MCF-7), and malignant carcinoma (KB).

### 6.4. Carotenoids

Carotenoids, found in fruits, vegetables, and algae, are the primary natural pigments used in the food industry due to their vibrant colors and positive biological effects [176], especially in reducing the risk of some chronic diseases [17,177]. For instance, *Dunaliella* and *Haematococcus pluvialis* are microalgae, which generate carotenes under stress circumstances, therefore being great carotenoid sources [177,178,179]. Fucoxanthin is one of nature’s most abundant carotenoids, derived also mostly from brown macroalgae [32,180,181,182].

Several regulatory authorities, including the FDA and the EFSA, have classified carotenoids as Generally Recognized as Safe (GRAS) [32]. These natural pigments, particularly β-carotene, can be added directly to the food matrices as in oils and butter to obtain a yellowish color [176] or to fortify the food in terms of pro-vitamin A activity [183]. The addition of 20% carrot flour to pasta (“vegetable-added pasta”), addition to the function of the colorant, also showed a higher antioxidant capacity and a higher total fiber compared to pasta without the addition of carrot flour [180]. Also, carotenoids responsible for the orange–yellow hues (e.g., bixin, lutein, and crocin) are used as colorants in ice creams [179] and in the beverage sector, such as lutein from marigold flowers [181].

Indirectly, carotenoids can be added to animal feeding for pigmentation and nutritional value enhancement of final products, such as in eggs or fish flesh [184,185], that will be subsequently consumed by humans or animals [176]. Aquaculture is one of the main industries that applies carotenoids in its products, such as β-carotene as a source of provitamin A, to enhance the immune system of fish, promote their growth, and prevent lipid peroxidation [186].

Carotenoids can also be used in packaging materials to promote food preservation, for example, β-carotene and/or lycopene to prevent the color modifications of food due to harmful processes, such as UV-induced damage or oxidation [187]. In addition, carotenoids can be used to extend the shelf-life of the product and, subsequently, their nutritional values by adding these natural pigments in protective films [187].

Possible health benefits can also be acquired from carotenoids. For instance, these pigments hold provitamin A activity. Vitamin A is a micronutrient that consists of a group of fat-soluble substances, such as retinol, and it regulates cell growth and differentiation, also playing an important role in immunological function, eye development, and vision improvement. It cannot, however, be metabolized within the human organism, and must be obtained from the ingestion of foods containing, for instance, carotenoids, especially β-carotene and β-cryptoxanthin, which promote its synthesis [50,188]. Carotenoids’ health benefits are overall related to their antioxidant, immuno-modulatory, and anti-inflammatory properties, and improvement of cognitive function, among others [50,189,190]. Carotenoids are also known to protect against some forms of cancer, since they inhibit abnormal cell proliferation, and can prevent skin cancer by mitigating UV radiation-induced damage [191,192]. There is also evidence that lycopene is related to the counteraction of prostate cancer, as it has been discovered an inverse association between prostate cancer and lycopene intake [193,194]. Other research has shown a correlation between lycopene levels and the reduction in hepatocellular lesions [195,196]. Carotenoids may help prevent heart disease, for instance, by decreasing the oxidation of low-density lipoproteins or preventing their synthesis, by disrupting gap–junctional communication, or by preventing the aberrant growth of cancer cells and working against certain forms of cancer [196,197]. Additionally, there is a new perspective on the function of carotenoids and their derivatives that links these chemicals to the regulation of the accumulation of body fat and adipocyte biology, which may have implications for the treatment of obesity [198,199]. Epidemiological studies have linked high circulation levels of β-carotene and other carotenoids to a decreased risk of metabolic and cardiovascular diseases, owing mostly to a high intake of fruits and vegetables [200]. Given that adipose tissue is an important deposit of carotenoids and retinol, and that body fat is a determining factor in susceptibility to many metabolic disorders, the potential health benefits of carotenoids and retinoids may be closely linked to the modulation of associated phenomena to “adiposopathy” fat [201]. Moreover, lutein accumulates in the cortices and membranes of the brain, thereby protecting the cognitive functions of older adults [202]. It is also reported that consuming spinach, cabbage, and egg yolk increases the amount of lutein and zeaxanthin in the retina, which helps prevent age-related macular degeneration [203]. In addition, there is evidence that correlates cognitive performance with the antioxidant activity of carotenoids [87]. For instance, a study stated that the long-term β-carotene treatment with a 50 mg intake every other day helped preserve cognitive performance in a healthy population [197]. Therefore, carotenoids hold several bioactivities with important health benefits.

Despite carotenoids demonstrating promising food industry applications with associated relevant health benefits, the direct application of these natural pigments is limited by their low stability. In this way, the application of micro- and nanoencapsulation technologies is being developed to overcome this issue [55,109]. Nonetheless, further research is needed within this area to optimize their stability and effectiveness.

### 6.5. Betalains

Betalains possess significant potential for enhancing foods through their pigmentation, antioxidant, antimicrobial properties, and other health-related bioactivities [204,205]. Nevertheless, their application in industrialized products, as the other mentioned pigments, faces hurdles due to maintaining their chemical stability, impacting their effectiveness as antioxidants and natural colorants [206].

The first FDA-approved betalain was betanin derived from red beetroot (*Beta vulgaris*). Betanins are utilized in a variety of products, including confections, ice cream, yogurt, ready-made frostings, cake mixes, and drinks [32]. Numerous studies have explored betalains’ potential as natural additives in food, highlighting critical factors, such as betalains’ profile, food matrix, and storage conditions for stability and acceptability [207]. For instance, Attia et al. found that betalains from red beet extract are reliant on concentration and share properties with synthetic red colorants for product acceptance [207]. Similarly, Kumar et al. showcased the stability and enhancement of betalain-infused banana spread, emphasizing their potential to prolong shelf life and enhance sensory experience in various food products [208].

The antioxidant potential of betalains in various foods has been investigated extensively. Studies by Attia et al., Coria-Cayupán & Nazareno, and da Silva et al. [207,209,210] demonstrate their efficacy in inhibiting lipid oxidation in corn oil, dairy products, and pork meat, respectively, suggesting betalains as viable natural alternatives to synthetic antioxidants while considering their sensory impact on food products.

The demand for sustainable food packaging solutions has led to the development of smart packaging utilizing biopolymers and natural extracts [211]. Betacyanins, such as those found in beetroots and cactus pears, have been utilized in pH-sensitive films capable of monitoring food freshness, as observed by Jamróz et al., Qin et al., Hu et al., and Yao et al. [212,213,214,215], indicating their potential for innovation in seafood packaging due to their color-changing properties and antimicrobial effects.

As mentioned in previous sections, betalains have been linked to a variety of biological activities, including anti-inflammatory, antiproliferative, and antimicrobial properties, as well as free radical scavenging, DNA-damage inhibition, gene control, and the prevention of lipid peroxidation [20,64,216,217,218]. Therefore, in vivo studies suggest that supplements based on these natural pigments may be effective for disorders associated with dyslipidemia, oxidative stress, and inflammation (e.g., hypertension, cancer, dyslipidemia, or stenosis of the arteries, among others). Furthermore, betalain from beetroot has been shown to improve exercise performance [100,219,220,221].

## 7. Conclusions and Future Trends

In recent years, significant events, such as global warming, the COVID-19 pandemic, and wars and conflicts, have prompted a reconsideration of new strategies to combat the adverse effects to which society has been subjected.

Thus, it is crucial not only to contemplate the reuse of existing resources, such as by-products rich in natural pigments, but also to explore extraction techniques applicable at an industrial scale, aiming to reduce process costs and promote environmental sustainability. However, the utilization of natural colors in food systems remains limited due to technical challenges. Exploring alternative colorant sources, such as agro-food by-products, is essential to achieve greater stability, physical–chemical viability, and enhanced color observation from both traditional and novel sources. Emerging methods, such as ohmic heating and PEF, offer the benefit of using less energy and water for chemical extraction. Additionally, these methodologies can readily induce electroporation in the food matrix, boosting production yields to over 80%. Moreover, new technologies must be adjusted to provide ecologically sustainable and cost-effective pigments from new and existing sources. Considering the lack of risk assessment studies, further toxicological research should be conducted, including an examination of the influence of different extraction methods to determine the safety of these natural pigments on biological systems.

Overall, this review provides a significant and thorough exploration of the bioactivities of natural pigments, their associated health advantages, and applications in the food domain, considering a zero-waste approach.

## Figures and Tables

**Table 2:** Pigments recovered from food by-products and their bioactivities.

By-Products	Pigments		Bioactivities	Results	References
Blackberry residues	Anthocyanins	Cyanidin-3-*O*-glucosidase, cyanidin-3-*O*-rutinoside, cyanidin-3-*O*-malonyl-glucoside, cyanidin-3-*O*-dioxalyl-glucoside	Antioxidant activity	DPPH assay: (98.05–113.60 µmoL TE/g dp); FRAP: (154.93–188.48 µmoL TE/g dp).	[111]
Grape by-products		Delphinidin-3-*O*-glucoside, cyanidin-3-*O*-glucoside, petunidine-3-*O*-glucoside, peonide-3-*O*-glucoside, malvidin-3-*O*-glucoside	Antioxidant activity	ORAC assay: 2.02–2.34 g/100 g ascorbic acid equivalent.	[84]
			Antimicrobial activity	Antimicrobial potential against *Yersinia enterocolitica*, *Pseudomonas aeruginosa*, *Salmonella enteritidis*, *Staphylococcus aureus*, and *Bacillus cereus.*	
Eggplant peel		Total anthocyanins	Antioxidant activity	Higher reducing power (39 ± 2.5 mg QE/100 g DE) and scavenging activity (IC_50_ = 2.88 ± 0.02 mg/mL).	[112]
Raspberry pomace		Cyanidin-3-*O*-sophoroside, cyanidin-3-*O*-glucoside, cyanidin-3-*O*-rutinoside	Antioxidant activity	The highest ability to neutralize DPPH radicals was IC_50_ = 8.15 mg FW/mL.	[108]
			Anti-inflammatory activity	Strong COX-2 inhibitory properties. The highest inhibitory activity was IC_50_ = 0.87 mg FW/mL.	
*Spirulina* sp.	Phycobiliproteins	Phycocyanin	Antioxidant activity	Dietary supplementation reduces the oxidative stress in liver and kidney induced by a diet enriched with lipid peroxides in Wistar strain rats.	[109]
***Spirulina platensis***		C-phycocyanin	Anti-inflammatory activity	Reduces micturition frequency and bladder inflammation in mice with cyclophosphamide-induced cystitis by inhibiting COX-2 and prostaglandin E receptor 4.	[113]
Porphyra haitanensis		Phycoerythrin	Anti-neurodegenerative activity	Inhibits the precursor protein of BACE1 and, therefore, reduces accumulation of amyloid-β precursor protein.	[114]
Cucumber and watermelon peels	Chlorophylls	Chlorophyll	Antioxidant activity	Antioxidant activity when compared to the control sample without extract addition.	[115]
Melon juice, pulp, peel, and seeds		Chlorophyll a and b	Antioxidant activity	Chlorophylls were only detected in peels.	[116]
Shrimp process by-products	Carotenoids	Astaxanthin, astaxanthin esters, and other carotenoids	Antioxidant activity	DPPH-scavenging activity of crude extract and its fractions were in the range of 3.08–3.74 mg TBHQ equivalent/mg of sample.	[117]
Cantaloupe waste		Lutein, β-carotene, violaxanthin	Antioxidant activity	The concentration needed to reduce IC_50_ was 7.33 μg/mL. This concentration is lower than that of β-carotene and Trolox standards (350 and 102.34 μg/mL, respectively).	[118]
Tomato peels		Lycopene, phytoene, phytofluene, β-carotene, cis-lycopene and lutein	Antioxidant activity	Induction period of sunflower oil was increased by increasing the concentration of carotenoids.	[119]
Acerola pulp and residue		Total carotenoids	Antioxidant activity	Pulp had greater antioxidant activity.	[120]
Red dragon fruit peels	Betalains	Total betalains	Antioxidant activity	ABTS assay: encapsulated betalains possessed higher antioxidant capacities (195.39–201.76 μmol Trolox/g microparticles).	[110]
			Anti-inflammatory activity	Duck embryo chorioallantoic membrane (CAM) vascular irritation assay showed that the anti-inflammatory activity of encapsulated betalains was five- to six-fold higher than that of non-encapsulated betalains.	
			Antigenic activity	Glutathione S-transferase (GST)-inducing activity of betalains was likewise improved four- to five-fold.	
*Opuntia* spp. peels		Betaxanthins (Indicaxanthin isomer I and II), Betacyanins (Betanidin-5-*O*-β-sophoroside, betanidin-5-*O*-β-glucoside, isobetanin, gomphrenin, betanidin)	Antioxidant activity	DPPH scavenging activity: 1.96–4.6 mg/mL extract.	[121]
			Antifungal activity	A higher activity performance of the extract was attained on *Trichoderma viride* and *Penicillum ochrochloron* strains.	
			Antimicrobial activity	The hydroethanolic extract exhibited effect on 6 of the 8 strains tested, being *Micrococcus flavus* and *Escherichia coli* the only resistant strains.	
Beetroots		Betanidin 5-glucoside, isobetanidin 5-glucoside, 2,17-bidecarboxy-neobetanin, 2-*O*-glucosyl-betanin, 17-decarboxy-betanidin, neobetanin	Antiulcer activity and anti-inflammatory activity	Betalain administration at doses 200, 400, and 800 mg/kg decreases the ulcer areas (UA) and index (UI); increases the curative index (CI) by 78.1, 78.4, and 78%, respectively; ameliorates the pathological damage induced by ethanol; prevented the decrease in gastric mucus content (116%) and reduced the stress oxidant; decrease in gastric mucosa thiobarbituric acid reactive species (TBARS) (28%) and mucus juice pepsin (56%).	[122]
*Opuntia stricta* pulp and peel (var. *Dillenii*)		Betalain-rich extract (BRE)	Gastroprotective activity	BRE supplementation (800 mg/kg) from pulp and peel to rats with ethanol-induced gastric ulcer significantly reduced: volume of gastric secretion (VGS) decreased by 35% and 34%, respectively; UI by 41% and 68%, respectively; and the curative radio (CR) by 41% and 68%, respectively, as compared to untreated ethanol-induced gastric ulcer.	[123]

## Data Availability

No new data were created or analyzed in this study. Data sharing is not applicable to this article.
